# District-level explanations for supporter involvement in political parties: The importance of electoral factors

**DOI:** 10.1177/1354068817699171

**Published:** 2017-03-28

**Authors:** Justin Fisher, David Cutts, Edward Fieldhouse, Bettina Rottweiler

**Affiliations:** Brunel University London, UK; University of Birmingham, UK; University of Manchester, UK; University College London, UK

**Keywords:** campaign activity, explanations for activism, political parties, supporters (non-members)

## Abstract

Traditional analyses of grass roots involvement in political parties have focussed almost exclusively on formal members. However, recent analyses across a range of democracies have shown that non-members (supporters) are playing important roles within political parties, including election campaigning, candidate and leader selection, online policy deliberations and even policy formation. The growing literature on this topic suggests that the involvement of supporters may be a function of party structure and availability of online recruitment. Using new data collected at the 2015 British general election, this article extends the examination of supporter involvement but challenges these assumptions. It shows that supporter activity is better explained by responses to electoral factors and that the focus on online recruitment seriously underplays the enduring importance of human contact.

## Introduction

In recent years, clear evidence has emerged from several countries that traditional notions of party membership have come under challenge, with non-members or supporters playing key roles in party activities conventionally associated with formal party members (Cross and Gauja, 2014a, 2014b; Fisher et al., 2014; Gauja, 2015a, 2015b, 2015c; Gauja and Jackson, 2016; Hazan and Rahat, 2010; Kosiara-Pedersen et al., 2012; Mjelde, 2015; Rahat et al., 2014; Sandri and Seddone, 2015; Scarrow, 2015, Webb et al., 2016). Parties are finding it increasingly difficult to maintain membership organizations and, in response to declining membership rates, are experimenting with new organizational styles to develop links with supporters – non-members (Scarrow, 2015). This has manifested itself in a variety of ways: involvement in election campaigning, candidate and leader selection (including primaries), online policy deliberations and even policy formation, leading one author to propose a framework to catalogue these developments, which rests on distinct boundaries in respect of what activities supporters may or may not participate (Mjelde, 2015). This goes well beyond Duverger’s (1954) concentric circles of increasing affiliation and participation, such that the boundaries between supporters and members are increasingly indistinct. The causes of this are various. Rahat et al. (2014: 4) argue in the Israeli case, for example, that dealignment between voters and parties is accelerating the opening of opportunities for supporters, whereas Sandri and Seddone (2015: 25) also point to declining party membership and the declining importance of cleavage politics. Indeed, Gauja (2015b: 90) suggests that this may reflect a shift in political parties, defining and organizing themselves ‘in terms of individual citizens rather than group interests’.

Scarrow (2015), however, argues that it is a deliberate strategy. Parties are blurring the lines or boundaries between members and supporters; partly in response to voter disaffection, with primaries in particular being used to indicate a ‘break from the past’ (Sandri and Seddone, 2015: 29); but also as a function of the availability and use of new technologies, which makes it easier to link supporters with parties. Gauja (2015b), for example, identifies examples in Australia and Britain, whereby parties have utilized online technology to facilitate policy discussions beyond the parties’ traditional memberships. Certainly, the web in particular is a means by which supporter activity can be facilitated, whether through volunteering, donating money or ultimately joining the party (Scarrow, 2015: 148). Along similar lines, Mjelde (2015: 300) suggests that this openness to supporters may be a function of societal and technological changes transforming the nature of campaigning from labour to capital intensive, thereby reducing parties’ need for formal members. There is an appealing logic to this argument, but it is at odds with findings in Britain, at least, which demonstrate that labour-intensive grass roots campaigning delivers stronger electoral payoffs than those that incur cost (Fisher, 2011; Fisher et al., 2014).

Notwithstanding, organizational changes in the British Labour Party are an excellent example of such broader developments, with supporters being required simply to pay a fee of £3 to take part in the election of the party’s leader in 2015; a development analogous to the selection of the leader of the Partito Democratico in Italy (Kenig, 2009), and the selection of French Socialist Party’s presidential candidate in 2011, where supporters of the Parti Socialiste and the Parti Radical de Gauche were required to pay €1 in order to participate (Fisher et al., 2014: 77). Such processes can create what Scarrow (2015) describes as fluid affiliation categories within multi-speed membership parties. She argues that parties can pursue three main strategies to boost participation: increase the rewards associated with traditional membership, reduce the costs of joining a party, and change or redefine what membership or enrolment means. Parties that adopt all three would be pursuing a multi-speed approach to membership, which both bolsters traditional membership, but also creates opportunities for supporter involvement (Scarrow, 2015: 128). The key difference with such new affiliation categories compared with traditional membership is that they are low cost, do not require long-term commitments and offer immediate opportunities for participation and communication. In return, parties gain invaluable contact information, which can be used to help nurture engagement and possibly future membership (Scarrow, 2015: 135–136). Supporters also deliver further advantages for parties by being less ‘sensitive to the traditional party discourse’ than members, thereby allowing parties more flexibility (Sandri and Seddone, 2015: 26).

Election campaigning is a particularly important area in terms of supporter activity. Rahat et al. (2014: 30) find, for example, that in the Israeli case, parties make recruiting supporters before elections a top priority, whereas Fisher et al. (2014) demonstrate in the British case that supporter involvement in election campaigns is extensive. Analysing district-level campaigns in the 2010 general election, they show that a significant proportion of campaigns (around three quarters) at the district or constituency level involved supporters (Fisher et al., 2014). Not only that, the participation of supporters was nontrivial. On average, supporters engaged in around two-thirds of the activities undertaken by members. The principal variation revolved around high- and low-intensity participation – supporters were much less likely to engage in pre-election voter contact: doorstep canvassing and telephone contact. Indeed, in some activities such as leaflet delivery, the evidence reflected the experience in Australia where the distinction between members and supporters, in respect of core campaigning activities, is ‘essentially meaningless’ (Cross and Gauja, 2014b:12). Yet supporters were not simply additional workers, duplicating the activities of members. While supporter activities did complement those of members, they also supplemented them. In sum, Fisher et al. (2014) showed that supporters made independent and positive contributions to all three main parties’ campaigns.

Supporters then clearly matter to many political parties in a variety of democracies. However, far less work has been done in respect of explaining supporter recruitment and the level of activities in which they engage. In other words, the extant literature says relatively little about variation between parties in terms of levels of supporter recruitment or supporter activities within parties. These questions matter because just as with more conventional party members, there are sometimes very significant variations between parties, which require explanation. Not only that, it is important to understand why, as with party members, there may be variation in respect of low-or high-intensity activities. In this article, therefore, we seek to address two important questions:

- What explains supporter recruitment?
- What explains levels of supporter activity?

## Explaining supporter recruitment and activity

The existing literature suggests two broad explanations for variation in supporter recruitment: the structure of a party – its position in the party system and to an extent, its traditional ideological profile – and the electoral fortunes of a party, with electoral popularity being a catalyst for recruitment. The emphasis in much of the existing literature is on the former. Gauja and Jackson’s (2016) study of the Australian Greens, for example, suggests that a party such as this, which is part of a broader social movement, may be more likely to recruit supporters than ‘mainstream’ political parties. The same may have been true of the Liberal Democrats in Britain, remembering that prior to entering the coalition government in 2010, the party shared some of the characteristics of the Australian Greens in as much as they were somewhat outside the mainstream and often the recipient of votes from electors who had previously supported the main two parties. Thus, Fisher et al. suggest that the Liberal Democrats’ success in recruiting supporters in the 2010 British general election campaign may have reflected the party’s traditional and ideological commitment to community politics and the similar ideological structuring of Liberal Democrat members and supporters (Fisher et al., 2014: 81; Whiteley et al., 2006: 65). A variant of this thesis is also outlined by Mjelde (2015: 306). He suggests that older parties may be less willing to accommodate supporters, especially where the party is more hierarchically structured. His analysis here is based on radical right parties, but the point about hierarchy may well be relevant, when comparing more and less established parties.

**Figure 1.**
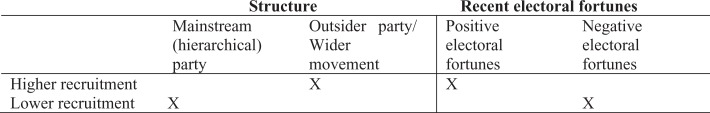
Explanations of supporter recruitment.

An alternative hypothesis to the structural model, however, is also put forward by Fisher et al. (2014). Born of a lack of previous data by which they could assess the extent to which their findings in 2010 were typical, they suggest that parties might find it more difficult to recruit supporters where a party had little chance of electoral success or where the election outcome was very predictable (Fisher et al., 2014: 92). From this perspective, supporters would be more likely to be active in tighter elections, and where a party’s possible chances of success are fairly strong. Such reasoning is informed theoretically by rational choice, such that pivotality (or at least a perception of pivotality) is a key driver for participation. Empirically, too, there is evidence that member retention is influenced strongly by electoral fortunes (Fisher, 2000; Fisher et al., 2006). Variations in supporter recruitment from this perspective are a function of electoral fortunes and supporters’ responses to them rather than the structural or ideological positioning of a party. We summarize these two perspectives in Figure 1.

If structure is a persuasive argument, we should observe differentiation in recruitment between ‘mainstream’ and ‘outsider’ parties. ‘Outsider’ parties should recruit supporters more extensively as they are more likely to be part of a broader social movement and/or they are less hierarchical, and these variations should hold over time. However, if electoral fortunes are a better explanation of variation, we should observe change over time depending on an individual party’s electoral performance. Thus,

**H1**: Supporter recruitment will be more extensive in outsider parties than in mainstream ones.
**H2**: Supporter recruitment is a function of electoral popularity, where reduced popularity leads to a decline in supporter recruitment and vice versa.

An associated aspect of this question is the efforts made by parties themselves, which relates to how supporters are recruited. The existing literature suggests that the availability and promotion of online engagement is a strong cue (Gauja, 2015b; Mjelde, 2015; Scarrow, 2015). Scarrow’s analysis (2015: 148–151) is particularly detailed and ranks both countries and party families (capturing the ideological profile of a party) in respect of their online accessibility for members, supporters and donors. In respect of volunteers, Australia, Canada and the United Kingdom are comfortably the most advanced in terms of volunteer accessibility, whereas in terms of party families, there is little difference in respect of volunteer accessibility (though centre-right and ‘others’ score marginally higher). The reliability of those scores is, however, amplified when accessibility overall is assessed, with Social Democrats, Liberals and Greens scoring noticeably higher. Supporter recruitment can therefore be characterized as varying both in terms of structural factors (the type of party in terms of ideological profile), but also, in part, as a function of parties’ own efforts from a top-down perspective, with parties that create more online opportunities being more successful in recruiting supporters.

However, while the findings in the extant studies are helpful, they suffer from a lack of comparison with other modes of recruitment. In other words, the assumption is that if online facilities for recruitment exist, they must be influential. But mobilization can take a number of forms alongside online. An alternative hypothesis is therefore required. Fisher et al. (2016) show, for example, that in terms of electoral turnout, voters respond much better to personal contact than parties’ online mobilization efforts. The same logic may also apply, therefore, in terms of the means by which supporters are recruited – namely that personal contact will be more effective. We therefore posit three hypotheses:

**H3**: Supporters are more likely to be recruited online rather than offline.
**H4**: Modes of recruitment will vary depending on whether a party is a mainstream one or an outsider.
**H5**: Centre-left parties will be more successful in recruiting online, due to higher levels of accessibility.

Our focus now becomes explanations of the level of election activity undertaken by supporters. Previous work on party supporters has shown that the efforts made in campaigns are nontrivial and make an independent and positive impact upon the strength of campaigns (Fisher et al., 2014). However, there is variation in terms of the range of activities undertaken by supporters and indeed in comparison with party members, supporters in both the British and Italian cases are generally more likely to engage in leaflet delivery, taking numbers at polling stations and helping out in the campaign office – low-intensity activity, and less likely to involve themselves in contacting electors prior to the election – high-intensity activity (Fisher et al., 2014, Sandri and Seddone, 2015: 40; Seyd and Whiteley, 1992, 2002; Whiteley et al., 1994; Webb et al., 2016).

Broadly speaking, we can identify three explanations in the literature to explain variation. Once again, there are those rooted in party structure and electoral fortunes. From these perspectives, we would expect variation to occur between mainstream and outsider parties, or as a function of a party’s electoral standing – once again, better electoral standing should be an incentive for great levels of supporter activity. Our hypotheses are therefore as follows:

**H6**: The type of supporter activity undertaken is a function of party structure.
**H7**: The type of supporter activity undertaken is a function of electoral popularity.

However, there is also a third possible explanation in respect of existing party strength. Here, we would expect variation in supporter activity in the first instance to be a function of the existing strength of local parties, with greater strength promoting supporter activity. Previous research suggests varying results for the impact of existing party strength. Fisher et al. (2014) showed that, in 2010, levels of supporter activity were in part functions of existing local party strength but that there was significant variation by party. The relationship between existing party strength and levels of supporter activity was much stronger in the case of the Liberal Democrats and much weaker for both the Conservatives and Labour. This would suggest some traction for the broad party structure thesis, since in 2010, the Liberal Democrats could be classed as more of an outsider party.

To test this thesis further, we seek to assess whether or not existing party strength is a factor in the number of activities undertaken by party supporters and also test for electoral fortunes thesis by analysing the impact of the previous election results on levels of subsequent supporter activity. Thus,

**H8**: The level of supporter activity will be greater where there is existing party strength.
**H9**: The level of supporter activity for individual parties will be larger in districts where electoral competition is greater for that party.

## Data and measurement

In order to test these rival hypotheses, we use both new data collected at the British general election of 2015 together with similar data collected at the 2010 election – see Fisher et al. (2014). The data are derived from surveys of the election agents of Conservative, Labour, Liberal Democrat, Scottish National Party (SNP), Plaid Cymru (PC) and United Kingdom Independence Party (UKIP) candidates who stood for election in districts (constituencies) in Great Britain (632 maximum). Election agents are responsible for the organization and conduct of a candidate’s campaign and are therefore extremely well placed to comment on the recruitment of supporters and their involvement in campaigns. Questionnaires were set to all agents of the candidates from parties under examination immediately after polling day.^1^ Responses to the 2015 study comprise of 244 Conservative, 336 Labour, 332 Liberal Democrat, and 204 UKIP agents in Great Britain, and 31 SNP (maximum 59) and 21 PC (maximum 40) in Scotland and Wales, respectively.^2^ All of these parties either had a long history of participating in national elections and winning seats on a regular basis, or in the case of UKIP had the potential to do so, with the party fielding candidates in almost all seats in Great Britain and having enjoyed significant success in both opinion polls and the European elections in the preceding years.^3^

The survey contained a number of questions related to supporter involvement in the parties’ campaigns, including whether or not supporters were recruited, the number recruited, the means by which they were recruited and the campaign activities in which they took part (with identical questions for party members for comparison). Five campaign activities were identified through binary (YES/NO) response categories – delivering leaflets; taking numbers at polling stations; helping at the campaign office, telephoning electors and doorstep canvassing. Identical questions (with the exceptions of those on recruitment methods) were included in a comparable election study at the 2010 election.

These data allow us therefore to assess whether there are variations in supporter recruitment across six separate parties and test which explanation (structure or electoral fortunes) is more persuasive, since we can compare results over two elections (2010 and 2015) rather than at just one time point. In this respect, Britain is an excellent case by which to examine the structure versus electoral fortunes perspectives. First, there is the existence of both mainstream and outsider parties, who regularly engage in electoral activity in a relatively stable party system. Thus, although our testing applies to parties in only one country, the method and party structure in Britain lends itself to replication in other countries. To test the structure hypothesis, therefore, we compare supporter recruitment by the main GB parties (the ‘mainstream’ parties) with the national parties (SNP and PC) and UKIP – the ‘outsider’ parties.

Second, in the two elections under consideration (2010 and 2015), there was considerable variation on electoral fortunes for some parties under examination; while for others, there was little change. This makes the British elections of 2010 and 2015 ideal to test the electoral fortunes thesis. In both elections, the circumstances for recruiting active supporters for the Conservatives and Labour were arguably strong. The opinion polls suggested that both elections would be tight, with the possibility that either party could form a government (albeit most likely in a coalition or as a minority government) (Cowley and Kavanagh, 2016: 232). In the case of the Liberal Democrats, however, the picture in 2015 was very different from the one in 2010. From soon after entering a governing coalition with the Conservatives following the 2010 election, the party’s popularity plummeted, and repeated poor performances in local authority and European elections after 2010 further highlighted the party’s electoral plight (Cutts and Russell, 2015). Under these circumstances, we would expect Liberal Democrat supporter recruitment to be lower in 2015 compared with 2010, since the incentives for participation would be less attractive for individuals who had not committed themselves to formal party membership. Equally, in Scotland, the SNP’s prospects were radically different in 2015 compared with 2010. Following the referendum of Scottish independence in 2014, the SNP’s polling figures surged despite the referendum rejecting independence (Mitchell, 2015). In Wales, by way of contrast, PC’s electoral prospects in 2010 and 2015 were very similar (Bradbury, 2015). Thus, we would expect a growth in SNP supporter recruitment, with that of Plaid being unchanged. For all parties except UKIP, we have data for both elections, which allows us to confirm whether the results hold over two data points.

## Results

We assess hypotheses 1 and 2 (the recruitment of supporters) in Table 1, which illustrates the proportion of district (or constituency) campaigns recruiting supporters in 2010 and 2015 and the mean numbers of supporters recruited where this occurred.^4^ We assess the structure hypothesis first, where we expect supporter recruitment to be greater in outsider parties (hypothesis 1). The evidence is not supportive. In both 2010 and 2015, the outsider parties were less likely to recruit supporters than the mainstream ones. This also applies in respect of the numbers of supporters recruited on average. In only one instance do outsider parties outperform the mainstream ones – the mean number of SNP supporters recruited compared with Labour in 2015. If we classify the Liberal Democrats as outsiders in 2010, there is a little more support, but as is clear in 2015, that success was not repeated.

**Table 1.** Supporter recruitment by party.

	% Recruiting party supporters (2010 in parenthesis)	Mean numbers recruited (2010 in parenthesis)
Conservative	65 (75)	22 (22)
Labour	74 (75)	15 (13)
Liberal Democrat	45 (86)	24 (19)
Scottish National Party	58 (67)	18 (6)
Plaid Cymru	43 (29)	12 (12)
United Kingdom Independence Party	51 (–)	6 (–)

*Note*: *n* for percentage recruiting party supporters: Conservative = 157; Labour = 246 Liberal Democrat = 147; SNP = 18; PC = 9; UKIP = 101; *n* for mean number of supporters recruited: Conservative = 152; Labour = 237; Liberal Democrat = 144; SNP = 18; PC = 9; UKIP = 98.

The electoral fortunes thesis fares better (hypothesis 2). The proportion of districts recruiting supporters for Labour is virtually identical (as is the mean number of supporters recruited), whereas for the Conservatives, there is a small decline – 65% of districts compared with 75% in 2010, though the mean number recruited is unchanged. For the Liberal Democrats, however, the impact of the party’s unpopularity following the 2010 election is stark. In 2010, some 86% of district-level campaigns recruited supporters. In 2015, however, that proportion sank to 45% – a drop of nearly 50%. However, somewhat surprisingly, the average number recruited rose from 19 to 24. This suggests that supporter recruitment may have been better targeted compared with 2010 and indeed, the largest numbers of party supporters were recruited in the party’s nominally safest seats, which given that party’s perilous electoral position was where the party focussed most attention (Fisher et al., 2015). In respect of the other parties, the evidence for the electoral fortunes thesis is a little more mixed. On the one hand, while the SNP recruited supporters in fewer districts in 2015, despite the party’s popularity, the mean number recruited rose threefold. Equally, however, while PC recruited an identical number of supporters on average, the proportion of districts in which they were recruited increased.

On balance, therefore, there is more support for the electoral fortunes hypothesis than the structural one. Electoral fortunes were influential as predicted for Labour, the Liberal Democrats and to an extent the SNP. The slight fall in Conservative recruitment is possibly more difficult to explain. On electoral prospects alone, we would expect supporter recruitment to be maintained. In one sense, it is: nearly two-thirds of Conservative campaigns recruited supporters. But of course, compared with 2010, the level fell by ten percentage points. Clearly, an additional explanation is required. One possibility is the ‘cost of governing’ – popularity may wane among supporters the longer a party is in power, particularly if it has to make unpopular decisions. A better explanation, however, is one rooted in Fisher, Denver and Hands’ hierarchy of election outcomes (2006). In respect of the retention of party members, they argue that election performance matters and that some outcomes are better than others. Thus, winning is always better than losing, but that a new victory is in turn better than repeating a victory. In this case, the possibility of ending Labour rule in 2010 would have been a better recruiting sergeant for supporters than the prospect of simply maintaining Conservative rule, with a strong possibility that that would be in coalition, particularly given that no public polls suggested a Conservative majority was a likely outcome and only 11% of voters thought such an outcome likely.^5^ Overall then, it would appear that electoral fortunes and supporters’ reaction to them may explain supporter recruitment better than analyses based on parties’ structural position.

Hypotheses 3 to 5 (the modes of supporter recruitment) are examined in Table 2. Our data enable us to test the rival hypotheses as party agents were asked to identify the principal means were by which supporters were recruited in their district.^6^ What is very clear for all parties is that although online interaction does contribute to supporter recruitment, it has only a marginal impact – far less important than is implied in the extant literature. Overall, only 9% of agents reported recruiting supporters in this way. For all parties, two other means of recruitment are more significant: offline interaction (which includes responses to telephone calls and leaflets) and most obviously, human contact (either on the doorstep, in the street, through word of mouth or through friendship or familial links). These findings challenge the theses that focus solely on the role of technology. For sure, the availability on online facilities helps. But just as with campaigning, it is the human touch that delivers by far the most benefits (Fisher et al., 2014, 2016). In short, online matters; but human contact matters a great deal more. The first hypothesis (hypothesis 3) is not therefore supported.

**Table 2.** Means of supporter recruitment by party 2015.

%	Con	Lab	Lib Dem	Nat. (SNP/PC)	UKIP	All
Human contact	45	39	37	24	31	38
Online interaction	8	11	6	5	9	9
Offline interaction	17	19	21	10	13	18
Prior activists	6	8	9	14	0	7
Self-starters	9	12	6	38	21	12
Other	15	13	21	10	26	17
*N*	121	199	115	21	80	536

In respect of hypothesis 4, there is some support for the structure argument. For mainstream parties, human contact and offline interaction matters more than for outsider parties. Outsider parties, however, are more likely to attract self-starters (people who approach the party themselves, often through a party office), and national parties in particular are more likely to more likely to attract prior activists (those who had previously helped the party or a related single-issue campaign).

The ideological profile thesis (hypothesis 5) is not supported, however – there is no variation between centre-left and centre-right parties. However, electoral fortunes may play a part. Responses for the national parties are heavily skewed towards the SNP. Thus, 47% of SNP supporters were self-starters. In the context on the 2015 election, this mode of recruitment is therefore also partially explained by the party’s electoral fortunes.

In terms of recruitment then, the extant literature which emphasizes party structure and online accessibility as key drivers for supporter recruitment does not find great support when tested against alternative hypotheses. For sure, there are isolated examples which support the structure thesis, such as the number of SNP supporters recruited and the greater propensity for outsider parties to recruit ‘self-starters’. Equally, online recruitment does matter for all parties. But, in general, rival hypotheses perform better. The likelihood of supporter recruitment is better explained by parties’ electoral fortunes, and human contact matters a great deal more than online in terms of bringing supporters on board. Potential supporters are much more likely to respond to the electoral prospects of a party and the greater accessibility of human contact.

The nature of election activities in which party supporters engage is shown in Table 3 and allows us to test hypotheses 6 and 7. Here, we see some support for the party structure thesis (hypothesis 6). Supporters of UKIP and PC were generally less active than supporters assisting the mainstream parties across a range of categories with the exception of UKIP activity in campaign offices. However, the counter evidence here lies with the SNP, where supporter activity resembles that of the mainstream parties to much greater degree. SNP supporters were at least as likely as Conservative and Labour supporters to engage in doorstep canvassing (in fact, slightly more likely). These patterns for the SNP in 2015 add further weight to the electoral fortunes thesis (hypothesis 7). Across all but one activity (telephoning electors) SNP supporters engaged in significantly more activity compared with 2010 – especially in respect of doorstep canvassing. And in that activity, we see further support for the electoral fortunes thesis – Liberal Democrat campaigns were less likely to have supporters involved in 2015 compared with 2010. However, in other respects, there is less support for this thesis, with Liberal Democrat supporters more likely to deliver leaflets, although in other activities there was barely any change from 2010. Overall, therefore, our hypotheses produce mixed results. The structure thesis (hypothesis 6) finds support for some outsider parties but not others, whereas the electoral fortunes thesis (hypothesis 7) finds strong support for the positive impact of improved electoral prospects, but only support for the negative aspects in respect of the most high-intensity area of activity – doorstep canvassing.

**Table 3.** Activities of supporters.

% saying YES (2010 in parenthesis)	Cons (*n* = 153)	Lab (*n* = 242)	Lib Dems (*n* = 140)	SNP (*n* = 19)	PC (*n* = 11)	UKIP (*n* = 103)
Delivering leaflets	100 (92)	97 (89)	98 (94)	100 (82)	91 (42)	90
Polling station number takers	54 (65)	34 (33)	47 (47)	32 (20)	9 (18)	17
Helping at campaign office	43 (54)	51 (56)	39 (40)	72 (43)	18 (9)	100
Telephoning electors	20 (24)	25 (27)	16 (16)	21 (20)	9 (0)	4
Doorstep canvassing	51 (42)	45 (38)	14 (22)	53 (19)	27 (33)	10

We now look to test hypotheses 8 and 9 by assessing the extent to which existing party strength explains the levels of activity undertaken by supporters and comparing these effects with those of the electoral status of a district or constituency in respect of that party. We also assess whether these patterns hold when we examine only high-intensity activity.

To capture levels of supporter activity (the dependent variable), we create a single additive scale of the five election participation items shown in Table 3.^7^ The impact of existing party strength and electoral fortunes on levels of supporter activity is tested using a zero-truncated Poisson model. We select this technique because it is used to model count data for which the value of zero cannot occur (since supporters will have engaged in at least one activity). The additive scale ranges from one to five activities, so it is not possible to score a zero.^8^ We confine our analyses to the three major parties in this case, as the relatively small number of cases for the national parties makes extensive analysis impossible.

Our independent variables capturing party strength are the size of the local district membership and the proportion of the district covered by an active local party. In order to test the electoral fortunes hypothesis, we add further independent variables capturing the marginality of the seat for the individual party following the results of the 2010 general election. There are four categories of seat: ultra-marginal, where the winning margin after the 2010 general election was less than 5%; marginal, where the winning margin was between 5% and 10%; safe, where the relevant party held the seat with a winning margin of more than 10%; and not held (or ‘hopeless’), where the relevant party did not win a seat and was more than 10% behind the winning party. We create binary variables for the first three categories thus making ‘not held’ the reference category. We hypothesize that supporters will be more active in seats where the contest is closer (ultra-marginal or marginal seats) or in seats where there is a good chance of victory (safe seats).

In terms of party strength (hypothesis 8), the findings in Table 4 suggest that a larger number of party members are positively associated with more supporter activity for the Liberal Democrats and Labour, whereas neither of the two measures of party strength have a statistically significant effect for the Conservatives. The results in Table 4 also indicate support for the electoral fortunes thesis (hypothesis 9). It is clear that the electoral status of a seat drives supporter activity with the strongest positive effects found in the ultra-marginal seats. Even so, all three categories (ultra-marginal, marginal and safe) prompt more supporter activity overall for the Conservatives, Labour and Liberal Democrats than in their not held (or hopeless) seats.

**Table 4.** Existing membership strength, previous electoral performance and supporter activity – zero-truncated Poisson.

Dependent variable = level of supporter activity	Conservative (*n* = 153)			Labour (*n* = 242)			Lib Dems (*n* = 140)		
	*b*	SE	Significance	*b*	SE	Significance	*b*	SE	Significance
Constant	0.386	(0.122)	**	0.609	(0.069)	**	0.149	(0.096)	ns
No. of party members	0.116	(0.071)	ns	0.078	(0.037)	**	0.114	(0.052)	**
% covered by active local	–0.016	(0.052)	ns	0.081	(0.047)	ns	−0.001	(0.080)	ns
Ultra-marginal seat	0.877	(0.159)	**	0.460	(0.111)	**	0.747	(0.227)	**
Marginal seat	0.740	(0.161)	**	0.408	(0.111)	**	0.710	(0.209)	**
Safe seat	0.599	(0.151)	**	0.245	(0.112)	**	0.727	(0.217)	**
Log likelihood	−228.898			−358.924			−179.057		

*Note*: SE: standard error; ns: not statistically significant. Number of party members and % covered by active local organization are standardized.

**p < 0.01; *p < 0.05.

To ease interpretation, we can transform these coefficients into incidence rate ratios.^9^ For instance, being a Conservative ultra-marginal, holding the other variables constant in the model, increases the level of supporter activity by a factor of 2.40 than that of not held (hopeless) seats, or equivalently, it increases the expected number by 140% when compared against the reference category (not held).^10^ Similar incidence rate ratios are recorded for Labour and Liberal Democrat ultra-marginals. For Labour, the level of supporter activity increases by a factor of 1.50 (+50%), while for the Liberal Democrats, it rises by a factor of 2.11 (+111%) when compared against not held (hopeless) seats.

We can also calculate the predicted amount of supporter activity in Conservative, Labour and Liberal Democrat ultra-marginals where the other marginality types are held at zero and the other two variables (number of party members and percentage actively covered by the local organization) are held at their means. The predicted amount of supporter activity is highest in Conservative closest contests at 3.51, compared with 2.92 and 2.48 for Labour and the Liberal Democrats in their most marginal seats. The overall patterns then are clear – electoral circumstances have a far more consistent effect on the level of supporter activity than existing party strength.

We extend these analyses by focussing solely on high-intensity activity (defined as participation in voter contact either on the doorstep or by telephone). Here, we use a dichotomous or binary dependent variable where supporter involvement in high-intensity activity is coded as 1 and non-involvement as 0. The independent variables are identical to those in Table 4 and the binary nature of the dependent variable means that logistic regression is used. The results are shown in Table 5. They show that for all three parties, it is electoral circumstances that drive levels of supporter activity. Measures of party strength for all parties fail to reach statistical significance. By way of contrast, all categories of seat boost activity for Labour and the Liberal Democrats, whereas the same is true for marginal seats for the Conservatives.

**Table 5.** Existing membership strength, previous electoral performance and supporter involvement in high-intensity activity.

Dependent variable = supporter involvement in high-intensity activity	Conservative (*n* = 153)			Labour (*n* = 242)			Lib Dems (*n* = 140)		
	*b*	SE	Significance	*b*	SE	Significance	*b*	SE	Significance
No. of party members	0.35	(0.35)	ns	0.10	(0.22)	ns	0.15	(0.25)	ns
% covered by active local org.	−0.26	(0.23)	ns	0.01	(0.17)	ns	−0.10	(0.33)	ns
Ultra-marginal seat	1.25	(0.65)	ns	1.32	(0.50)	**	2.43	(0.96)	**
Marginal seat	1.59	(0.61)	**	0.97	(0.45)	*	1.93	(0.83)	*
Safe seat	0.86	(0.53)	ns	1.01	(0.38)	**	2.08	(0.88)	*
Constant	−0.51	(0.35)	ns	−0.44	(0.20)	*	−2.26	(0.36)	**
McFadden’s *R*^2^		0.06			0.06			0.17	
Log likelihood		−99.38			−158.00			−61.12	

*Note*: ns: not statistically significant.

**p < 0.01; *p < 0.05.

**Figure 2.**
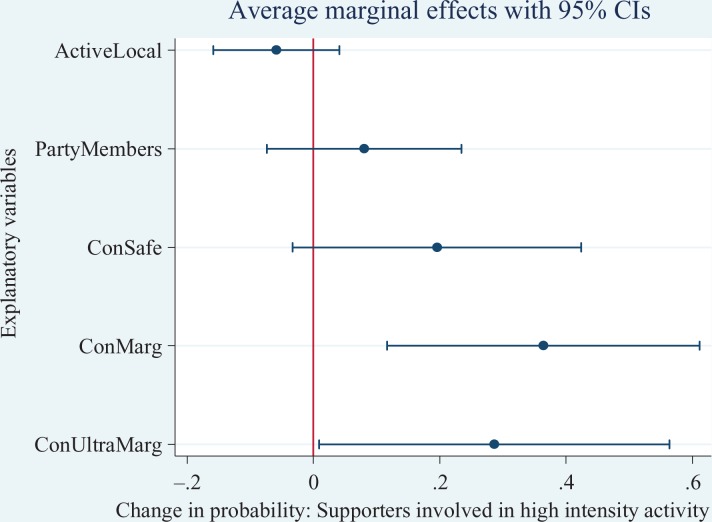
Average Marginal Effects (with 95% confidence intervals) of Conservative supporters participating in high-intensity activities.

**Figure 3. fig3-1354068817699171:**
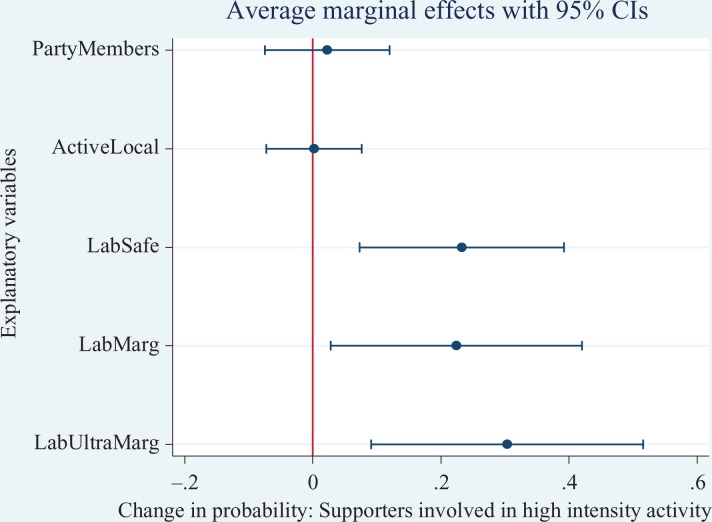
Average Marginal Effects (with 95% confidence intervals) of Labour supporters participating in high-intensity activities.

To ease interpretation, we estimate the discrete change on the probability for each of the values averaged across the observed values. These average marginal effects (AMEs) are graphically illustrated in Figures 2 [Fig fig3-1354068817699171]to 4. For the Conservatives, on average, the probability of supporter involvement in high-intensity activities is 36 percentage points higher in marginal seats than in Conservative not held (or ‘hopeless’) seats. Similarly, Labour supporter involvement in higher intensity activities is on average 30 percentage points in ultra-marginals and 22 and 23 percentage points in marginal and safe seats higher than in not held (hopeless) Labour seats. And, like Labour, the probability of Liberal Democrat supporter involvement in high intensity activities is higher, on average, in ultra-marginal seats (33 percentage points) than marginal (27 percentage points) and safe (28 percentage points) when compared with not held (hopeless) Liberal Democrat seats.

**Figure 4.**
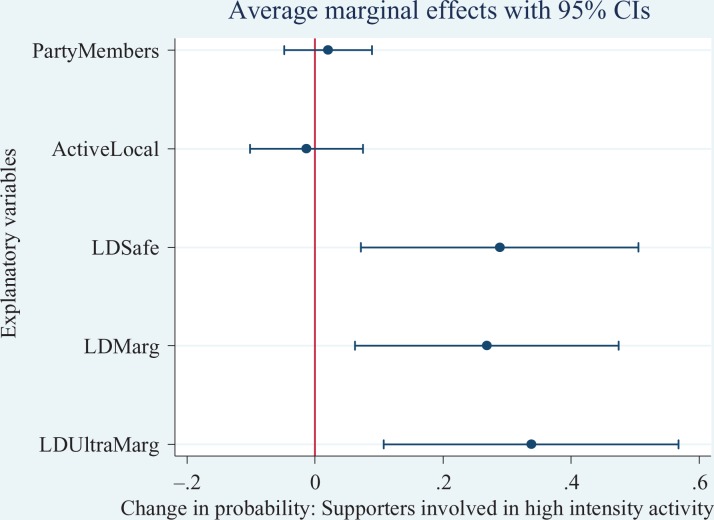
Average Marginal Effects (with 95% confidence intervals) of Liberal Democrat supporters participating in high-intensity activities.

In sum, we find some support for hypothesis 9 but weaker support for hypothesis 8. Existing party strength is only relevant in isolated cases and has no impact on participation in high-intensity activity (hypothesis 8). By way of contrast, the level of party competition in the district influences both the overall level of supporter activity and the propensity to engage in high-intensity activity (hypothesis 9). Supporters are more active in seats where the contest is close or where there is a good chance of victory.

## Conclusions

Supporters are an increasingly integral aspect of parties’ activities in a range of democracies. Not only is participation widespread, the level of activity is clearly nontrivial. In election campaigns, we observe that although supporters tend to be more likely to engage in low-intensity activity, their contribution remains important. And, there is growing evidence that supporters are becoming integrated into other aspects of party organization.

The implications of such developments are numerous. First, it challenges our traditional understanding of parties, whereby formal members constitute the principal source of voluntary labour and electorates for internal decision-making. Second, it challenges models of party organization, which have focussed on membership incentives through participation in a range of party processes (e.g. Duverger, 1954; Strom, 1990). Third, it challenges the party decline thesis, whereby formal membership decline is a key indicator, with a party evolution approach, recognizing newer modes of ‘membership’ (Cross and Gauja, 2014a; Gauja, 2015b; Gauja and Jackson, 2016; Sandri and Seddone, 2015). Summing up the importance of supporters, Gauja (2015c: 233) argues that party membership has traditionally been viewed as a ‘static concept’ when in fact, we should view it instead as an evolving one, reflecting how parties are able to accommodate differing expectations and norms of both the state and citizen’s changing preferences in terms of participation. It is clear that members are not the only source of activism and as such models of party organization that focus on participation incentives based on the assumption that formal members singularly constitute the grass roots need to be re-cast. As Gauja (2015c: 232) notes, while ‘party decline is a prominent theme in the scholarly literature…citizens are looking to alternate means of political expression’. Parties are evolving and adapting and sometimes almost apocalyptic suggestions in the party decline literature need revisiting.

But key questions emerge in respect of the recruitment of supporters. Is supporter recruitment a function of party structure, or is it better explained by responses to parties’ electoral fortunes? Although much of the existing literature suggests that structural factors are likely to be paramount, our testing of alternative explanations suggests that electoral fortunes may offer a more convincing explanation – supporters are more or less likely to be recruited depending upon the electoral popularity of the party. Just as other work on party organization, which suggests that electoral performance can be a strong influence on member retention (Fisher, 2000; Fisher et al., 2006), so it is also true of supporters. By way of contrast, the structure thesis has far less support. And, these findings are borne out not only in terms of recruitment but also in terms of levels of activity. Electoral circumstances have a strong impact on levels of supporter activity, even when controlling for parties’ existing strength. Supporters are active in seats where the party is likely to win or where the contest is close.

This article therefore represents a direct challenge to the existing literature which places a strong emphasis on the importance of party structure. It also challenges other aspects of the extant literature in respect of the means by which supporters are recruited. It shows that the existing emphasis on online recruitment is over-stated, and when online is compared with other modes of recruitment, it is clear that – in Britain, at least – human contact remains the most potent recruiting sergeant. In sum, this article suggests that party scholars need to look beyond structural explanations when seeking to explain activism. Rather, they need to pay more attention to ones rooted more in rational choice. Activists directly consider the appeal of their involvement as a function of its likely impact – they back winners. And the reverse is also true – activity is diminished if a party is unpopular. All of which presents potential problems for parties, since the important role of supporters in their electoral campaigns can be affected seriously by their level of electoral popularity.

## Supplemental material

PPQ699171_Appendix - District-level explanations for supporter involvement in political parties: The importance of electoral factors Click here for additional data file. PPQ699171_Appendix for District-level explanations for supporter involvement in political parties: The importance of electoral factors by Justin Fisher, David Cutts, Edward Fieldhouse, and Bettina Rottweiler in Party Politics

## Notes

1. In 2015, no questionnaires were sent to agents in Buckingham (the Speaker’s seat) or to Rochdale and Heywood and Middleton. In the case of the latter two constituencies, the local authority (Rochdale) did not publish details of the agents. No electoral agent address details were available for 18 UKIP agents. This was principally the case where the agents were also parliamentary candidates. The responses were representative of the total population of agents for the three GB parties, based on the electoral status of their seats (see Online Appendix).

2. Figures from the 2010 study are taken from Fisher et al. (2014).

3. Up to a point, the same was true in respect of the Greens, who won a seat in 2010 and stood in 573 seats compared with 335 in 2010. However, unlike UKIP, the party did not experience the same level of momentum in terms of opinion poll ratings or election successes.

4. In a small number of cases (5), the reported number of supporters declared was considered to be unreliable, being far in excess of other responses and adversely distorting the mean. As a result, those were removed from calculation of the mean. For 2015, this represented three Labour cases, one Liberal Democrat and one UKIP case. As a result, the mean number of supporters may be a slight underestimate.

5. Voter election outcome expectations are derived from an Ipsos-MORI poll in April 2015. https://www.ipsos-mori.com/researchpublications/researcharchive/2565/Expected-general-election-outcome-19792005.aspx. Accessed 11 August 2015.

6. In these tests, we combine the two national parties (SNP and PC) as there were fewer responses from these parties to the relevant question.

7. In order to maximize the number of cases in the model featured in Table 4, multiple imputation was used for the two independent variables, based upon the electoral status (level of party competition) of the seat for the individual party.

8. As a comparison, we also ran an OLS regression model using the same additive scale as the dependent variable. The results from the OLS are largely similar to the zero-truncated Poisson models reported in the text with the key predictors significant in both. Only Labour safe seat is significant in Table 4 and not significant in at the 95% level in the OLS model, although it is significant at the 90% level. The full OLS results are shown in the Online Appendix.

9. The term incident rate ratios are used here because of the regression method employed. A rate is the number of events per time (or space), which is how our dependent variable is derived. The Poisson regression coefficients can be interpreted as the log of the rate ratio, whereas the rate at which events occur is called the incidence rate. Given that the difference of two logs is equal to the log of their quotient, it is also applicable to interpret the parameter estimate as the log of the ratio of expected counts (the ‘ratio’ in incidence rate ratios). So as an example, Conservative ultra-marginal has a coefficient of +0.887. The exponential of this is 2.40. So for Conservative ultra-marginals compared to non-held seats, while holding the other variable constant in the model, are expected to have an incidence rate of 2.40 times that of non-held seats.

10. This is calculated as follows: 100 × (2.40 − 1)% = +140%.
