# Supplementary material for: District-level explanations for supporter involvement in political parties: The importance of electoral factors
Source: Party Politics. 2017 Mar 28;24(6):743–54. doi: 10.1177/1354068817699171 (PMC6201166; doi:10.1177/1354068817699171)
Supplement: PPQ699171_Appendix - District-level explanations for supporter involvement in political parties: The importance of electoral factors [file PPQ699171_Appendix.docx]

Appendix

Table A1. Representativeness of Responses

| % | Ultra-Marginal  Maj. <5% | Marginal  Maj. 5-10% | Safe Held  Maj.>10% | Safe Not Held  Maj.>10% |
| --- | --- | --- | --- | --- |
| **All Seats (631)** |  |  |  |  |
| Conservative | 11 | 15 | 36 | 38 |
| Labour | 11 | 13 | 28 | 48 |
| Lib Dems | 5 | 6 | 5 | 84 |
| **Responses** |  |  |  |  |
| Conservative (244) | 7 | 13 | 43 | 37 |
| Labour (336) | 10 | 12 | 23 | 55 |
| Lib Dems (332) | 5 | 6 | 6 | 83 |

**Table A2.** Existing Membership Strength, Previous Electoral Performance and Supporter Activity - OLS

| *Dependent Variable =*  *Level of Supporter Activity* | **Conservative**  (n=153) | | | **Labour**  (n=242) | | | **Lib Dems**  (n=140) | | |
| --- | --- | --- | --- | --- | --- | --- | --- | --- | --- |
|  | b | S.E. | Sig | b | S.E. | Sig | b | S.E. | Sig |
| Constant | 2.000 | (.157) | ** | 2.232 | (.101) | ** | 1.693 | (.078) | ** |
| No. of Party Members | 0.289 | (.183) | n.s. | 0.194 | (.096) | ** | 0.258 | (.111) | ** |
| % Covered by Active Local Org | -0.039 | (.115) | n.s. | 0.147 | (.090) | n.s. | -0.027 | (.124) | n.s. |
| Ultra Marginal Seat | 1.621 | (.363) | ** | 0.908 | (.252) | ** | 1.057 | (.409) | ** |
|  |  |  |  |  |  |  |  |  |  |
| Marginal Seat | 1.239 | (.317) | ** | 0.756 | (.238) | ** | 0.993 | (.393) | ** |
| Safe Seat | 0.791 | (.250) | ** | 0.384 | (.206) | * | 1.018 | (.402) | ** |
| **Adj. R^2^** | .248 | |  | .183 | |  | .322 | | |

Note 1: ** p<.01 * p<.05 n.s not statistically significant; 2. Number of Party Members and % Covered by Active Local Organisation are standardised.
